# Protein-bound solute removal during extended multipass *versus* standard hemodialysis

**DOI:** 10.1186/s12882-015-0056-y

**Published:** 2015-04-18

**Authors:** Sunny Eloot, Wim Van Biesen, Mette Axelsen, Griet Glorieux, Robert Smith Pedersen, James Goya Heaf

**Affiliations:** Department of Nephrology, Ghent University Hospital, De Pintelaan 185, 9000 Ghent, Belgium; Institute of Public Health, Aarhus University, Nordre Ringgade 1, 8000 Aarhus C, Denmark; Flexdialysis Aps, Copenhagen, Denmark; Department of Nephrology, Herlev Hospital, University of Copenhagen, Copenhagen, Denmark

**Keywords:** Solute removal, Recirculation, Water consumption

## Abstract

**Background:**

Multipass hemodialysis (MPHD) is a recently described dialysis modality, involving the use of small volumes of dialysate which are repetitively recycled. Dialysis regimes of 8 hours for six days a week using this device result in an increased removal of small water soluble solutes and middle molecules compared to standard hemodialysis (SHD). Since protein-bound solutes (PBS) exert important pathophysiological effects, we investigated whether MPHD results in improved removal of PBS as well.

**Methods:**

A cross-over study (Clinical Trial NCT01267760) was performed in nine stable HD patients. At midweek a single dialysis session was performed with either 4 hours SHD using a dialysate flow of 500 mL/min or 8 hours MPHD with a dialysate volume of 50% of estimated body water volume. Blood and dialysate samples were taken every hour to determine concentrations of p-cresylglucuronide (PCG), hippuric acid (HA), indole acetic acid (IAA), indoxyl sulfate (IS), and p-cresylsulfate (PCS). Dialyser extraction ratio, reduction ratio, and solute removal were calculated for these solutes.

**Results:**

Already at 60 min after dialysis start, the extraction ratio in the hemodialyser was a factor 1.4-4 lower with MPHD versus SHD, resulting in significantly smaller reduction ratios and lower solute removal within a single session. Even when extrapolating our findings to 3 times 4 h SHD and 6 times 8 h MPHD per week, the latter modality was at best similar in terms of total solute removal for most protein-bound solutes, and worse for the highly protein-bound solutes IS and PCS. When efficiency was calculated as solute removal/litre of dialysate used, MPHD was found superior to SHD.

**Conclusion:**

When high water consumption is a concern, a treatment regimen of 6 times/week 8 h MPHD might be an alternative for 3 times/week 4 h SHD, but at the expense of a lower total solute removal of highly protein-bound solutes.

## Background

To overcome the non-physiological characteristics of standard 3 times/week 4 hour in-centre hemodialysis, alternative dialysis regimens are being developed, aiming to reduce labour costs and patient burden, and increase patient comfort and solute removal [1-14]. In addition, production of dialysate is being recognized as having an important financial and ecological cost, which is likely to even increase in the future [15]. There is an increasing interest in these alternative renal replacement therapies, whereby either duration of a single session (extended dialysis) [4,7,11,14], the frequency per week (frequent dialysis) [1,2,6,8,9,13] or a combination thereof [3,12] are different from standard 3 times/week dialysis. For most of these settings, regular dialysis monitors are used, with online production of dialysate. Some alternative approaches have been proposed, whereby either dialysate is prepared as a batch [16-18], or is available in industrially prepared bags [19].

Recently, multipass hemodialysis (MPHD) was described as a feasible, economic and ecologic alternative to deliver home hemodialysis [20]. A regime of daily nocturnal dialysis eight hours six times a week, using a dialysate bath of one half of the calculated total body water, i.e. 20-25 L, which is continually recycled at a dialysate flow of 500 mL/min, while blood flow is conventional (>200 mL/min) was proposed as treatment paradigm. Obvious advantages are the ease of use, and the low water consumption, making the technique ideal for home hemodialysis. Using this technique, a significantly higher weekly removal was obtained for small water soluble solutes and for middle molecules like β_2_-microglobulin, as compared to 3 times 4 h standard hemodialysis (SHD) [20]. Since higher middle molecule removal has been linked to reduced mortality [21], and due to the limited dialysate consumption, MPHD seems to be a very promising technique for performing (portable) home hemodialysis.

Protein-bound solutes are known as difficult to remove by conventional hemodialysis as the ligand proteins often have a molecular weight above or at the borderline of the cut-off of currently used high flux dialysis membranes. This lack of adequate removal may have important clinical consequences, since several protein-bound solutes have been linked to progression of renal failure, inflammation, vascular disease, and mortality [22-35].

The present study was set up to investigate removal of protein-bound solutes by 8 h MPHD as compared to 4 h SHD.

## Methods

### Patients

The patients and methods have been described in a previous publication [20]. Ten stable HD patients, all receiving standard in-centre HD three times per week were included in the original study, but, due to sample corruption, only nine are included in the present study. Exclusion criteria were: age less than 18 years, psychiatric disease, ultrafiltration requirement more than 4 L per session, possibility of pregnancy and severe comorbidity. Prior to the study, catheter/fistula recirculation was excluded using the indicator dilution technique and the Krivitski method (HD 01 plus, Transonic Systems, Ithaca, New York State). All patients gave written informed consent according to the Helsinki II declaration. The protocol was approved by the local ethics committee ‘Videnskabsetiske Komité for Hovedstads Region’ (identification number H-2-2009-082) and registered on ClinicalTrials.gov (identification number NCT01267760).

### Study design

Each patient was studied twice with a one week interval. The patients used either the Polyflux 170H (3 patients) or Polyflux 210H with a surface area of 1.7 and 2.1 m^2^, respectively (Gambro, Lund, Sweden) for both sessions. The reference treatment was standard hemodialysis (SHD) lasting 4 hours, with a dialysate flow of 500 mL/min. The dialysate was continuously collected in a chamber placed on electronic weighing scales. A blender was placed in the chamber to assure adequate mixing before sampling.

The alternative treatment was multipass hemodialysis (MPHD) using a dialysate chamber with a volume corresponding to 50% of the patient’s total body water (TBW), which was estimated as either 55% (female) or 60% (male) of dry weight. One patient, with a body weight of 111 kg and an estimated TBW of 66 L was only treated with 30.4 L due to the limitations of the dialysate chamber. The dialysate was prepared by the AK-200 dialysis machine (Gambro, Lund, Sweden), and was identical for both treatments. With MPHD, dialysate was recirculated via the chamber which was placed on electronic scales, and in which an oscillation mechanism was installed to assure optimal mixing. Two pumps controlled dialysate inflow (500 mL/min - UF/2) and outflow (500 mL/min + UF/2), and thus ultrafiltration (UF). Dialysate temperature was set at 36-37°C at the dialyser inlet, and was continuously registered.

Fractionated heparin was used as anticoagulation, at the patient’s usual dose (at the start of SHD and MPHD). After 4 hours MPHD a new similar bolus was administered.

### Sampling and analysis

Blood samples were collected from the inlet blood line at the start and at hourly intervals. In addition, one blood sample was taken from the blood outlet line at 60 min to obtain the dialyser extraction ratio. Blood samples were immediately centrifuged at 3000 rpm, after which the plasma was stored at −80°C until batch analysis. From the dialysate chamber, dialysate was sampled hourly and stored at −80°C.

Different protein-bound solutes were determined by high performance liquid chromatography (HPLC): p-cresylglucuronide (PCG) (molecular weight MW:284 Da, protein binding PB ~ 10%), hippuric acid (HA - 179 Da - PB ~ 50%), indole acetic acid (IAA - 175 Da - PB ~ 65%), indoxyl sulfate (IS - 213 Da - PB ~ 90%), p-cresylsulfate (PCS - 187 Da - PB ~ 95%). To determine the total concentration, serum samples were first deproteinized by heat denaturation [36] before HPLC analysis. IS and IAA (excitation λ_ex_: 280 nm; emission λ_em_: 340 nm) and PCS and PCG (λ_ex_: 265 nm; λ_em_: 290 nm) were determined by fluorescence analysis, and HA by UV detection at 245 nm [37]. Free fractions were determined according to Fagugli et al. [37] and serum total protein (TP) was analysed according to standard methods.

### Calculations

Reduction ratio (RR - %) of solutes was defined as a function of predialysis (C_pre_) and postdialysis concentrations (C_post_) of samples collected from the inlet blood line:

1 $$ \mathrm{R}\mathrm{R}\ \left(\%\right)=\frac{{\mathrm{C}}_{\mathrm{pre}}-{\mathrm{C}}_{\mathrm{post}}}{{\mathrm{C}}_{\mathrm{pre}}}\cdot 100 $$

During 8 h MPHD, RR was also calculated for the 0-240 min and the 240-480 min dialysis interval.

The dialyser extraction ratio (ER - %) was calculated as the relative change in concentration from the dialyser inlet (C_inlet_) towards the outlet (C_outlet_):

2 $$ \mathrm{E}\mathrm{R}\ \left(\%\right)=\frac{{\mathrm{C}}_{\mathrm{inlet}}-{\mathrm{C}}_{\mathrm{outlet}}}{{\mathrm{C}}_{\mathrm{inlet}}}\cdot 100 $$

Total solute removal (TSR - mg) at time point t was calculated from dialysate concentration in the chamber at that time point, and multiplied by either the volume of spent dialysate at time point t (SHD) or the dialysate volume in the chamber (MPHD). Total solute removal on weekly basis was calculated from TSR as measured and calculated in a single session and multiplied by the dialysis frequency per week.

Protein-bound solute concentrations at time point t were corrected for hemoconcentration by a factor (F) based on TP concentration predialysis versus time point t: F = TP_pre_/TP_t_. Likewise, dialyser outlet concentration (C_outlet_) was corrected by F = TP_inlet_/TP_outlet_.

### Statistical analysis

Data are expressed as mean ± SD assuming normally distributed populations. Statistical analyses were carried out using the parametric *t*-test for paired samples. A P ≤ 0.05 was considered to be statistically significant. All statistical analyses were performed using SPSS Statistics 21 (SPSS Inc, Chicago, IL) for Windows (Microsoft Corp, Redmond,WA).

## Results

The nine included patients (female n = 3) were 63.4 ± 12.7 years old and spent 7.1 ± 4.4 years on dialysis. Renal diagnoses were: hypertensive nephropathy (n = 3), polycystic renal disease (n = 1), glomerulonephritis (n = 1), chronic interstitial nephropathy (n = 1), and unknown (n = 3). Five patients had a residual daily diuresis of more than 300 mL/day. Patients dry weight was 79.8 ± 19.4 kg, resulting in a calculated TBW of 46.5 ± 11.2 kg. Accordingly, MPHD dialysate volume was 22.9 ± 5.0 L (range 13.8 to 30.4 L). Blood flow was 279 ± 43 mL/min during SHD and 279 ± 41 mL/min during MPHD (N.S.).

**Figure 1:**
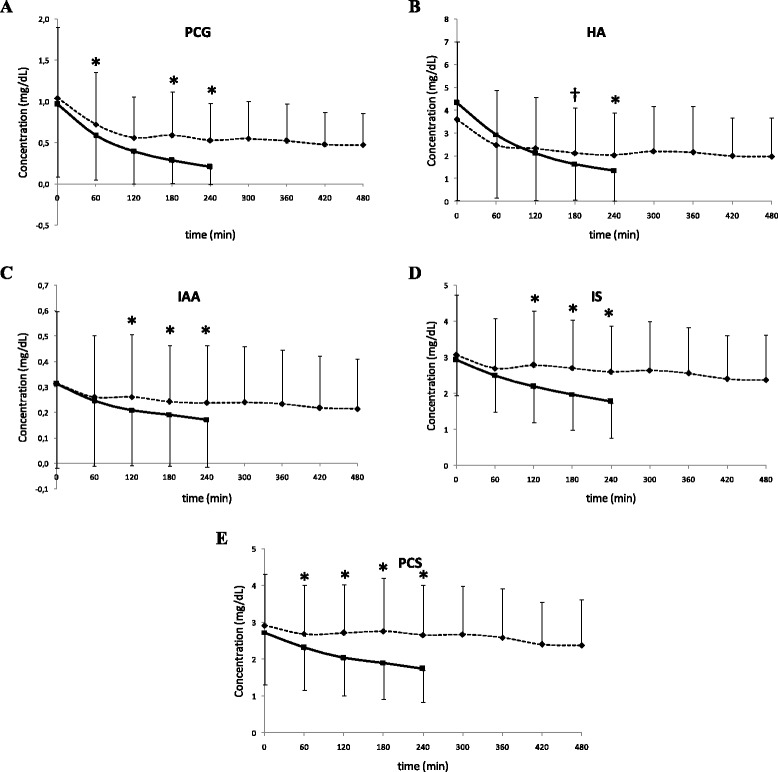
Serum concentrations at different time points during standard hemodialysis SHD (squares - full line) and multipass hemodialysis MPHD (diamonds - dotted line) for p-cresylglucuronide (PCG -panel ** A**), hippuric acid (HA - panel** B**), indole acetic acid (IAA - panel** C**), indoxyl sulfate (IS - panel **D**), and p-cresylsulfate (PCS - panel** E**). *P < 0.05 MPHD *versus* SHD. †P = 0.053 MPHD *versus* SHD.

Figure 1 illustrates the variation in serum concentration during SHD (squares - full line) and MPHD (diamonds - dotted line) for p-cresylglucuronide (PCG), hippuric acid (HA), indole acetic acid (IAA), indoxyl sulfate (IS), and p-cresylsulfate (PCS). Serum concentrations at start of the dialysis session were not different between both modalities in the individual patients (N.S.). The large standard deviations indicate important inter-patient variability in serum concentrations of these solutes. Nevertheless, it was in all patients consistently observed that concentration reductions were much smaller with 8 h MPHD as compared to 4 h SHD. In addition, it is noteworthy that during the second half of the 8 h MPHD, nearly no further change in concentration reduction was observed (Table 1).

**Table 1:** **Reduction ratios (%) for different protein-bound solutes in SHD and MPHD**

**Solute**	**SHD**	**MPHD**		
	**0-240 min**	**0-240 min**	**240-480 min**	**0-480 min**
Total PCG	81 ± 7	51 ± 5	9 ± 9†	55 ± 6*†
Total HA	68 ± 10	42 ± 7	1 ± 37†	43 ± 16*
Total IAA	46 ± 8	25 ± 5	9 ± 13†	32 ± 8*
Total IS	41 ± 11	14 ± 6	10 ± 12	23 ± 11*†
Total PCS	37 ± 9	10 ± 9	6 ± 18	16 ± 15*
Free PCG	84 ± 5	51 ± 5	10 ± 10†	56 ± 6*†
Free HA	78 ± 8	45 ± 11	−5 ± 38†	44 ± 16*
Free IAA	67 ± 28	33 ± 13	8 ± 21†	39 ± 18*
Free IS	59 ± 17	−7 ± 33	7 ± 43	−2 ± 55*
Free PCS	74 ± 11	−1 ± 44	7 ± 39	7 ± 51*

SHD: standard hemodialysis; MPHD: multipass hemodialysis; PCG: p-cresylglucuronide; HA: hippuric acid; IAA: indole acetic acid; IS: indoxyl sulfate; PCS: p-cresylsulfate.

*P < 0.05 *versus* SHD; †P < 0.05 *versus* MPHD_0-240min_.

Already at 60 min after dialysis start, a huge difference is observed between the extraction ratio for total and free fractions during SHD and MPHD, due to the recirculation in the latter modality (Table 2). Extraction in the hemodialyser for total concentrations of protein-bound solutes is a factor 1.4-1.8 (PCG, HA, IAA) and 3–4 (IS, PCS) larger with SHD as compared to MPHD, while for the free fractions of these solutes, it is a factor 1.4-1.6 (PCG, HA, IAA) and 2.1-2.5 (IS, PCS). Hence, there is an inverse correlation between extraction ratio and percentage protein binding in both SHD (R = −0.98) and MPHD (R = −0.99), but extraction of highly bound solutes like IS and PCS is even more hampered in MPHD compared to SHD.

**Table 2:** **Extraction ratios (%) at 60 min during SHD and MPHD for total and free fractions of different protein-bound solutes**

**Solute**	**SHD**	**MPHD**	**SHD/MPHD**
Total PCG	73 ± 15	53 ± 8*	1.4
Total HA	54 ± 10	31 ± 19*	1.7
Total IAA	29 ± 8	16 ± 8*	1.8
Total IS	13 ± 5	5 ± 5*	2.9
Total PCS	11 ± 6	3 ± 5*	3.9
Free PCG	78 ± 10	55 ± 9*	1.4
Free HA	65 ± 11	43 ± 8*	1.5
Free IAA	49 ± 23	31 ± 12*	1.6
Free IS	36 ± 9	17 ± 10*	2.1
Free PCS	38 ± 15	15 ± 10*	2.5

SHD: standard hemodialysis; MPHD: multipass hemodialysis; PCG: p-cresylglucuronide; HA: hippuric acid; IAA: indole acetic acid; IS: indoxyl sulfate; PCS: p-cresylsulfate.

*P < 0.05 versus SHD.

**Figure 2:**
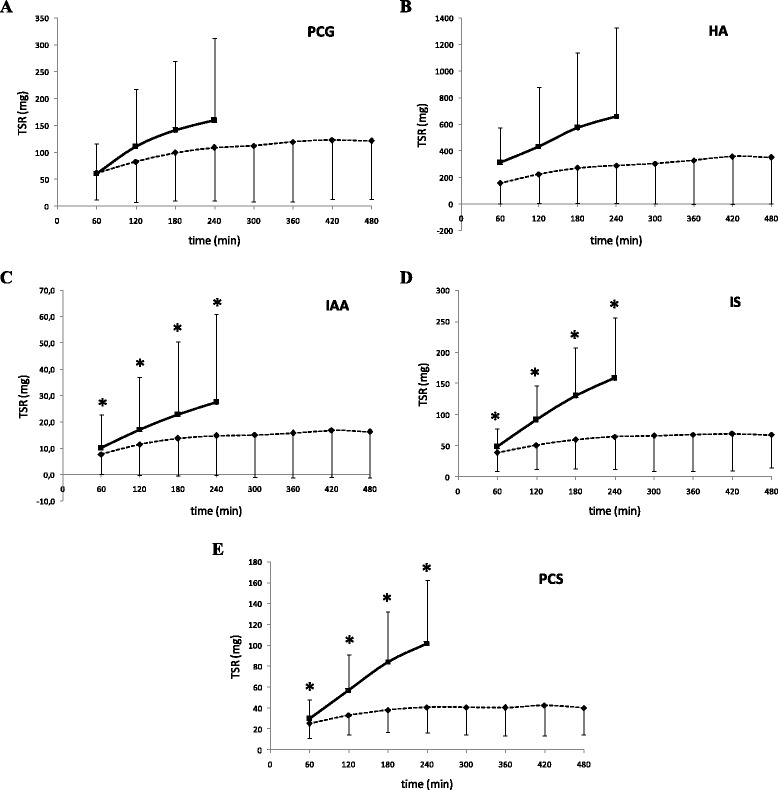
Cumulative total solute removal (TSR) at different time points during standard hemodialysis SHD (squares - full line) and multipass hemodialysis MPHD (diamonds - dotted line) for p-cresylglucuronide (PCG - panel** A**), hippuric acid (HA - panel** B**), indole acetic acid (IAA - panel** C**), and indoxyl sulfate (IS - panel** D**), p-cresylsulfate (PCS - panel** E**). *P < 0.05 MPHD *versus* SHD.

Figure 2 clearly indicates that the cumulative total solute removal (TSR) with MPHD *versus* SHD is, for all studied solutes, much smaller. Of note, TSR seems to saturate after 4 h MPHD. The normalized TSR for the amount of used dialysate was a factor 3–3.9 (PCG, HA, IAA) and 1.8-1.9 (IS, PCS) larger with MPHD as compared to SHD (Table 3), indicating the relative efficiency of the MPHD modality with regard to water use.

**Table 3:** **Total solute removal per litre used dialysate (mg/L) and the ratio of total solute removal during MPHD** ***versus*** **SHD**

**Solute**	**SHD**	**MPHD**		$\frac{{\mathbf{MPHD}}_{\mathbf{0}\mathbf{\hbox{-}}\mathbf{240}}}{\mathbf{SHD}}$	$\frac{{\mathbf{MPHD}}_{\mathbf{0}\mathbf{\hbox{-}}\mathbf{480}}}{\mathbf{SHD}}$
	**0-240 min**	**0-240 min**	**0-480 min**		
Total PCG	1.29 ± 1.22	4.22 ± 3.57	4.71 ± 3.93*†	3.6 ± 1.4	3.9 ± 1.7
Total HA	5.35 ± 5.39	10.4 ± 9.2	12.5 ± 11.3*†	2.7 ± 1.3	3.0 ± 1.0
Total IAA	0.22 ± 0.27	0.52 ± 0.44	0.57 ± 0.50	3.4 ± 1.5	3.7 ± 1.9
Total IS	1.29 ± 0.79	2.43 ± 1.72	2.54 ± 1.76*	1.8 ± 0.6	1.8 ± 0.7
Total PCS	0.83 ± 0.50	1.60 ± 0.87	1.56 ± 0.92*	2.0 ± 0.9	1.9 ± 0.9

SHD: standard hemodialysis; MPHD: multipass hemodialysis; PCG: p-cresylglucuronide; HA: hippuric acid; IAA: indole acetic acid; IS: indoxyl sulfate; PCS: p-cresylsulfate.

*P < 0.05 *versus* SHD; †P < 0.05 *versus* MPHD_0-240min_.

When results of the experimental single session were extrapolated to a weekly basis, the absolute TSR during 6 times 8 h MPHD *versus* 3 times 4 h SHD was larger for PCG and IAA (Table 4), not different for HA and smaller for IS and PCS. For 6 times 4 h MPHD *versus* 3 times 4 h SHD, TSR is only larger for PCG while smaller for IS and PCS, and for 6 times 2 h MPHD *versus* 3 times SHD, TSR is significantly smaller for HA, IS, and PCS.

**Table 4:** **Weekly total solute removal (mg) for 3 times 4 h SHD, 6 times 2 h MPHD, 6 times 4 h MPHD, and 6 times 8 h MPHD for different protein-bound solutes**

**Solute**	**3x4 h SHD**	**6x2 h MPHD**	**6x4 h MPHD**	**6x8 h MPHD**
Total PCG	480 ± 458	498 ± 452	655 ± 592*	729 ± 650*†
Total HA	1985 ± 2010	1338 ± 1296*	1738 ± 1688	2104 ± 2096†
Total IAA	83 ± 101	69 ± 70	89 ± 90	98 ± 105*
Total IS	478 ± 291	308 ± 232*	388 ± 314*	406 ± 320*
Total PCS	306 ± 183	199 ± 112*	246 ± 148*	241 ± 155**

SHD: standard hemodialysis; MPHD: multipass hemodialysis; PCG: p-cresylglucuronide; HA: hippuric acid; IAA: indole acetic acid; IS: indoxyl sulfate; PCS: p-cresylsulfate.

*P < 0.05 *versus* 3x4 h SHD; **P = 0.57 *versus* 3x4 h SHD; †P < 0.05 *versus* 6x4 h MPHD.

## Discussion

In this cross-over study comparing 4 h standard hemodialysis (SHD) with 8 h multipass hemodialysis (MPHD), concentration reduction and total solute removal were assessed for the protein-bound solutes p-cresylglucuronide (PCG), hippuric acid (HA), indole acetic acid (IAA), indoxyl sulfate (IS), and p-cresylsulfate (PCS). We found that already at 60 min after dialysis start, the extraction ratio in the hemodialyser was a factor 1.4-4 lower with MPHD *versus* SHD, resulting in lower reduction ratios and total solute removal. When TSR was calculated per litre of dialysate spent, MPHD appeared to be superior.

It was recently demonstrated that MPHD resulted in superior removal of small and middle molecular weight solutes when applied as 6 times/week 8 h dialysis, an effect that was already demonstrated previously using another batch system (Genius®, Fresenius Medical Care) [4]. Our current results add to this knowledge that MPHD applied in this regimen results in equal total solute removal for light and moderately protein-bound solutes, but lower removal for highly protein-bound solutes as compared to standard 3 times weekly hemodialysis. Most likely, this can be explained by the fact that only the free solute can pass across the membrane. As the serum concentration of free solute is rather low, and as the dialysate is recycled, there is a rapid equilibration between the concentrations of the free solute in the serum and in the dialysate. As a result, replenishment of the pool of free toxin in the serum, either by loosening from the protein or by rebound from other compartments, has no effect on solute removal, as the diffusive gradient with the dialysate is lacking. Indeed, we observe (Figure 1) that after some hours of dialysis, there is a plateau of the concentration in the dialysate, and accordingly, a significantly lower extraction ratio in the hemodialyser is observed already after 60 min. After this, no further solute removal is observed. The timing of this plateau phase comes more early as the solute is more strongly protein-bound. Extending the dialysis session is accordingly of no use to increase solute removal of protein-bound toxins when small volumes of dialysate are recycled.

For middle molecular structures such as β_2_-microglobulin, this diffusive transport over the dialysis membrane is much slower, so the equilibration/saturation is also occurring at a slower rate. An increase of dialysis time from 4 to 8 hours, maintaining the same amount of processed blood and dialysate, previously demonstrated a 81% higher β_2_-microglobulin removal, a phenomenon that was much less pronounced (26-36% increase) for the small water soluble solutes urea and creatinine [4]. This effect can be attributed to the slow transport of β_2_-microglobulin between the extraplasmatic and plasmatic compartment [38,39], resulting in a substantial rebound after conventional and short hemodialysis [40]. During extended dialysis, the solute is allowed more time to shift between compartments. Accordingly, extending the dialysis session allows the serum pool of the middle molecule to be replenished from other compartments, resulting in an increase in solute removal even when recycling the dialysate, as demonstrated previously [20].

A limitation of our study might be the mathematical extrapolation of the removal during a single session to the removal on weekly basis. Theoretically, since predialysis concentrations of toxins can be decreased in frequent dialysis, solute removal could be overestimated with frequent MPHD. However, as we are dealing with protein-bound solutes, only the free fraction can be eliminated during the dialysis session such that total removal is very low. Hence, it is likely that this free fraction is completely restored during the interdialytic interval, which was also found by Fagugli et al. investigating removal of protein-bound solutes in short daily dialysis (6 times weekly 2 hours) as compared to standard hemodialysis (3 times weekly 4 hours), even with a regular dialysis monitor and dialysate flow, so that saturation of dialysate could not play a role [37].

It could be hypothesized that in order to increase solute removal of protein-bound toxins, one needs to extend total weekly treatment time, and use sufficiently high ratio of dialysate to blood flow. A recent kinetic analysis supported this hypothesis [41]. If it is intended to use recycling of dialysate, an absorbent should be added to the dialysate system to keep the concentration of the free fraction of protein-bound solutes as low as possible.

Although it appears that MPHD is less suitable than SHD to remove protein-bound solutes, the advantage of MPHD is mainly the relatively higher amount of solute removed per litre of dialysate (Table 3). This makes the multipass system a suitable alternative to apply in the setting of daily extended dialysis at home [42], where the classical setup implies substantial technical modifications and results in a high consumption of water and electricity. The economy in water consumption is 33% (240 L/week *versus* 360 L/week), as more frequent dialysis is needed. Whereas this seems an impressive saving, we need to realize that water consumption is with 4-11% only a limited part of the carbon foot print of dialysis treatment, as compared to 35.7% for pharmaceuticals and 23.4% for medical equipment.

## Conclusion

MPHD appears to have some interesting features for use in the home setting, and results in more efficient use of dialysate. However, the currently proposed regimen of 6 times/week 8 hours seems to have no advantage over 3 times/week SHD in terms of removal of protein-bound solutes.

## Abbreviations

C: Concentration
ER: Extraction ratio
HA: Hippuric acid
HD: Hemodialysis
HPLC: High performance liquid chromatography
IAA: Indole acetic acid
IS: Indoxyl sulfate
MPHD: Multipass hemodialysis
N.S.: Non significant
PB: Protein binding
PBS: Protein-bound solutes
PCG: p-cresylglucuronide
PCS: p-cresylsulfate
RR: Reduction ratio
SHD: Standard hemodialysis (PBS)
TBW: Total body water
TP: Total protein
TSR: Total solute removal
UF: Ultrafiltration
